# Effects of Apigenin on Experimental Ischemia/Reperfusion Injury in the Rat Ovary

**DOI:** 10.4274/balkanmedj.2016.1386

**Published:** 2017-09-29

**Authors:** Zeynep Soyman, Sefa Kelekçi, Veysel Sal, Osman Şevket, Nihan Bayındır, Hafize Uzun

**Affiliations:** 1 Clinic of Obstetrics and Gynecology, University of Health Sciences, İstanbul Training and Research Hospital, İstanbul, Turkey; 2 Department of Obstetrics and Gynecology, İzmir Katip Çelebi University School of Medicine, İzmir, Turkey; 3 Department of Obstetrics and Gynecology, İstanbul University Cerrahpaşa School of Medicine, İstanbul, Turkey; 4 Department of Obstetrics and Gynecology, Bezmialem Vakıf University School of Medicine, İstanbul, Turkey; 5 Department of Histology and Embryology, Bezmialem Vakıf University School of Medicine, İstanbul, Turkey; 6 Department of Biochemistry, İstanbul University Cerrahpaşa School of Medicine, İstanbul, Turkey

**Keywords:** anti-Müllerian hormone, apigenin, ischemia/reperfusion injury, ovary, rat

## Abstract

**Background:**

Apigenin is a plant-derived compound belonging to the flavone class, which possess antioxidant, free-radical-scavenging and anti-inflammatory properties.

**Aims:**

To address the effects of apigenin on serum anti-mullerian hormone levels, tissue oxidative stress parameters and histopathological changes in ovarian ischemia/reperfusion injury.

**Study Design:**

Animal experiment.

**Methods:**

Twenty-eight female Wistar albino rats were randomly separated into four sections: Sham operation (group 1), ischemia/reperfusion plus saline (group 2), ischemia/reperfusion plus dimethyl sulfoxide (group 3) and ischemia/reperfusion plus apigenin (group 4). In all ischemia/reperfusion groups, a bilateral adnexal 3-h period of ischemia was performed, followed by 3-h of reperfusion. A single dose of 15 mg/kg apigenin was given intraperitoneally 60 min before reperfusion in group 4. After 3-h of reperfusion, both ovaries were removed, and blood samples were collected. The main outcome measures were serum anti-mullerian hormone levels, ovarian tissue malondialdehyde, total nitric oxide, Cu/Zn superoxide dismutase, catalase and glutathione levels and histopathological damage scores.

**Results:**

The ovarian tissue nitric oxide level was significantly lower, and the glutathione level was significantly higher in group 4 compared with groups 2 and 3. There was no significant difference in anti-mullerian hormone levels among the three ischemia/reperfusion groups. The histopathological damage score was lower in group 4 than in groups 2 and 3 (p>0.05).

**Conclusion:**

Administration of apigenin has no significant protective effect on ovarian reserve and tissue damage in ovarian ischemia/reperfusion injury.

Ovarian torsion is one of the most common gynaecological conditions. The ovary is partially or completely twisted around its vascular pedicle, compromising its blood supply. Ovarian torsion accounts for approximately 3% of gynaecological emergencies and is frequent in women of reproductive age (1). The nonspecific clinical presentation can lead to a delay in diagnosis that results in necrosis and loss of the ovary (2,3).

In the past, salpingo-oophorectomy without prior detorsion was the traditional mode of treatment, due to the fear of thromboembolism. Recent literature has demonstrated that conservative management of ovarian torsion by unwinding of the twisted adnexa can conserve ovarian function, irrespective of the colour of the ovary at the time of surgery (4). Ischemia/reperfusion (I/R) injury diminishes ovarian reserve, and detorsion of the ovary alone is insufficient to protect ovarian reserve (5).

Hypoxia caused by decreased blood flow leads to ischemic tissue damage. After detorsion, excess production of reactive nitrogen species (RNS) and reactive oxygen species (ROS) occurs in addition to accumulation of activated neutrophils with reperfusion precipitate reperfusion injury (6). The excessive production of RNS, ROS and their toxic products leads to cellular injury and lipid peroxidation in membranes.

Anti-mullerian hormone (AMH) is a dimeric glycoprotein produced by the granulosa cells of the growing preantral and small antral follicles (7). The blood AMH level has been shown to reflect the ovarian reserve (8). A decline of serum AMH levels has been shown in ovarian damage caused by I/R injury (5).

Apigenin is a plant-derived compound belonging to the flavone class, which possesses free-radical-scavenging, anti-inflammatory and antioxidant properties (9,10). Apigenin decreases the expression of inducible nitric oxide synthase (NOS) mRNA and protein, thereby inhibiting the synthesis of total nitric oxide (NOx) (9). Many studies have reported the beneficial influence of apigenin on I/R injury in brain (11), myocardium (12) and liver (13); however, there have been no studies indicating the influence of apigenin upon ovarian I/R injury thus far.

This paper aims to address effects of apigenin on serum AMH levels, ovarian tissue oxidative stress parameters and histopathological changes in ovarian I/R injury.

## MATERIALS AND METHODS

This project has been carried out in Bezmialem Vakıf University School of Medicine, Center of Experimental Animals. The study was approved by the Ethics Committee of Experimental Animals of Bezmialem Vakıf University (Approval number: 2015/54).

### Animals

Twenty-eight adult female Wistar albino rats varying in age from 14 to 16 weeks and in weight from 260 to 320 grams were used in the project. Animals were kept in cages with food and tap water ad libitium in a temperature-controlled environment at 22±2 °C and on a 12-h dark/light cycle. The oestrous cycle of each rat was determined by performing a daily vaginal smear. Rats with consistent 4-day cycles were involved in the study.

### Experimental design

Twenty-eight adult female Wistar albino rats were randomly separated into four sections with the use of computer-generated randomization (n=7); Sham operation (group 1), I/R plus saline (group 2), I/R plus dimethyl sulfoxide as a solvent (group 3) and I/R plus apigenin (group 4). The animals were anesthetized by using 75 mg/kg ketamine hydrochloride (Ketalar, Eczacıbaşı, Turkey) and 10 mg/kg xylazin hydrochloride (Rompun, Bayer, Turkey).

After anaesthesia, rats were placed in a dorsal recumbent position, and the abdominal skin was shaved and cleaned with the use of 10% povidone-iodine solution (Batticon; Adeka Laboratories). A 2.5-cm midline incision was carried out and the ovaries and uterine horns were examined. Rats in the sham group received no special treatment other than laparotomy and bilateral oophorectomy. In the other groups, a bilateral adnexal 3-h period of ischemia followed by 3-h of reperfusion were performed. The experimental ovarian I/R procedure was created by rotating the bilateral adnexa 360°, including the tubo-ovarian vessels, in a clockwise direction and then constrained to the abdominal wall by a 4-0 polyglycolic acid suture. A single dose of 0.5 ml of 0.9% NaCl and 0.3 mL of 0.9% NaCl + 0.2 mL DMSO were administered intraperitoneally 60 min prior to reperfusion in groups 2 and 3, respectively. Apigenin was administered as single dose of 15 mg/kg intraperitoneally 60 min prior to reperfusion in group 4. Apigenin (Santa Cruz Biotechnology Inc., Dallas, Texas, USA) was dissolved in 10% DMSO (Santa Cruz Biotechnology Inc., Dallas, Texas, USA) immediately before intraperitoneal injection. The administered dose of apigenin was based on the previous study (13). After a 3-h reperfusion period, both ovaries were taken away for histopathologic and biochemical examination.

After the procedures were finished, blood specimens were acquired by cardiac puncture from each rat for measurement of serum AMH levels. The rats were sacrificed by decapitation. Ovarian tissue damage was rated by histopathologic examination. All animals survived the procedure.

### Biochemical examination

### Tissue preparation and biochemical assay

Approximately 185-205 mg of each ovarian specimen was homogenized 20% w/v in 20 mM ice-cold Tris·HCl, pH 7.5 using a Bosch Scintilla SA (Switzerland). The homogenate was centrifuged at 5000 g for 11 min, and biochemical determinations were carried out in the supernatant fraction.

Lipoperoxidation was determined from the formation of malondialdehyde (MDA) according to the thiobarbituric acid method, with modifications (14). Thiobarbituric acid-reactive substance levels were determined using 1.55x10-5 M-1cm-1 as the molar extinction coefficient. Tissue NOx concentrations were evaluated colorimetrically as its stable metabolites, nitrate and nitrite, by the Griess reagent method using a commercial colorimetric assay (BioVision, Nitric Oxide Colorimetric Assay Kit, CA, USA). Tissue superoxide dismutase (SOD) (Cu/Zn SOD) levels were ascertained according to the method of Sun et al. (15) using inhibition of nitroblue tetrazolium (NBT) reduction with xanthine/xanthine oxidase as a superoxide generator. One unit of Cu/Zn SOD was described as the amount of protein that reduces the rate of NBT reduction by 50%. The tissue glutathione (GSH) concentrations were determined spectrophotometrically by the method of Beutler et al. (16). Serum AMH concentrations were determined by using a commercial enzyme-linked immunosorbent assay kit (MyBioSource, Rat AMH ELISA Kit Catalog No: MBS2509909, San Diego, California, USA).

### Histopathological examination

For histopathological analysis, ovarian tissues were fixed in 10% neutral buffered formaldehyde and embedded in paraffin. Cross-sections (5 μm) were obtained and stained with haematoxylin&eosin (H&E). Ovarian damage was evaluated by considering stromal congestion, oedema and infiltration, follicular degeneration and oedema and necrosis in the corpus luteum. The severity of these parameters was calculated as 0: absent, 1: minimal, 2: moderate and 3: severe (17). Sections were examined under light microscopy (Nikon Eclipse i5 light microscope with a Nikon DS-Fi1c camera and the Nikon NIS Elements version 4.0 image analysis systems. Nikon Instruments Inc., Tokyo, Japan). All tissue samples were evaluated by a single histologist who was blinded to the groups.

### Statistical analysis

Statistical analyses were carried out by using NCSS (Number Cruncher Statistical System) 2007 (Kaysville, Utah, USA). Categorical finding were examined by the Kruskal-Wallis test, and multiple comparisons were carried out by means of the Mann-Whitney U test. Descriptive statistical methods (mean, standard deviation) were used. The Kruskal-Wallis test was used in the case of non-normally distributed data. A Bonferroni-corrected Mann-Whitney U test was applied to dual comparisons of data that were established as significant by the Kruskal-Wallis test. A Mann-Whitney U test with Bonferroni corrected p values were used for the post hoc comparisons in Table 1. Results were considered significant at p<0.05.

## RESULTS

### Biochemical findings

The Table 1 lists the ovarian tissue MDA, NOx, Cu/Zn SOD, CAT, GSH levels, serum AMH levels and histopathological damage scores. group 2 and group 3 show greatly higher MDA and NOx levels and lower Cu/Zn SOD and GSH levels than the sham group (Mann-Whitney U test, p<0.05). There was no statistical difference in the CAT levels between the sham group and all I/R groups (Mann-Whitney U test, p>0.05). The NOx levels have been greatly lower in group 4 than in group 2 and group 3 (Mann-Whitney U test, p<0.05). The GSH levels have been greatly higher in group 4 than in group 2 and group 3 (Mann-Whitney U test, p<0.05). However, there was no statistical difference in the MDA levels between the group 4 and the other I/R groups. The Cu/Zn SOD levels were significantly higher in group 4 than in group 3 (Mann-Whitney U test, p<0.05). The NOx, CAT, Cu/Zn SOD, and GSH levels did not change significantly from group 2 to group 3 (Table 1).

The serum AMH concentrations were significantly lower in groups 2, 3 and 4 than in the sham group (Table 1). There was no significant difference in AMH levels among the three I/R groups.

### Histopathological findings

**FIG. 1.**
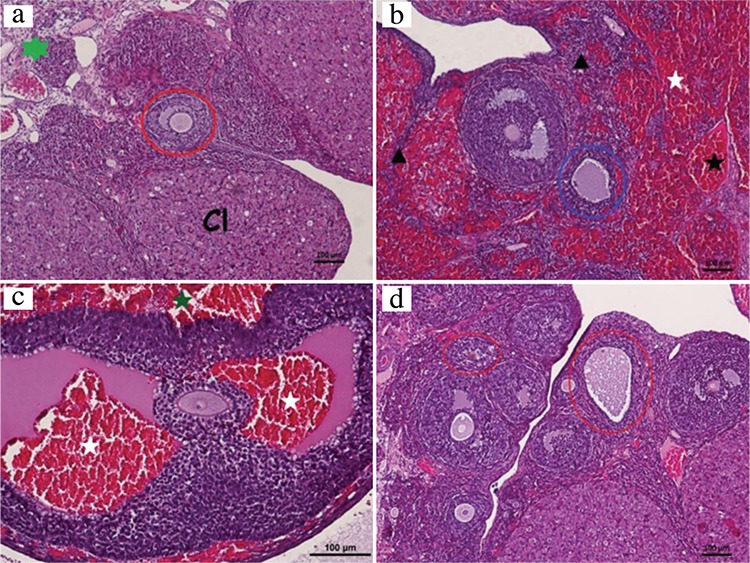
Group 1 (a) shows a normal histological appearance, Corpus Iuteum, a secondary follicle (lined by red) in the cortex are seen. The stroma is marked with a green star. Group 2 (b) shows haemorrhage (white asterisk), congestion (black asterisk), infiltration (black arrowhead) and follicular degeneration (lined by blue). Group 3 (c) shows follicular degeneration, haemorrhage within the follicular antrum (white asterisks) and the perifollicular area (green asterisk). Group 4 (d) shows follicular degeneration (lined by red lines) with vacuolisation in the granulosa cells and luteal cells, intercellular oedema in the granulose layer and perinuclear oedema in luteal cells. The absence of haemorrhage is noted.

The sham group showed a normal histopathological appearance (Figure 1a). Ovary tissues from both groups 2 and 3 showed some severe histopathological alterations, including follicular degeneration (including vacuolization, perinuclear oedema and degeneration in follicular granulosa cells and vacuolisation and apoptosis in corpus luteum cells), congestion, haemorrhage and infiltration (Figures 1b, 1c; respectively). Histopathological damage scores were higher in the all I/R groups as comparing with the sham group (Mann-Whitney U test, p>0.05). The histopathological damage score was lower in group 4 than in groups 2 and 3 (Mann-Whitney U test, p>0.05). In group 4, the severity of vacuolization and apoptosis in corpus luteum cells and of congestion and infiltration was decreased; additionally, no haemorrhage was observed. However, follicular degeneration, including vacuolization in granulosa cells, was obvious (Figure 1d).

## DISCUSSION

The present study demonstrated that apigenin treatment may not be effective in preventing ovarian I/R injury. Apigenin pretreatment partially reduced oxidative stress; however, it did not ameliorate ovarian reserve nor tissue damage. The accumulation of activated neutrophils that release ROS causes I/R injury. ROS lead to direct cellular injury (18). Antioxidant enzymes, such as Cu/Zn SOD, GSH and CAT, are responsible for protecting tissues against free radicals (11,19). Some experimental ovarian I/R injury models have reported that antioxidant agents such as erythropoietin, coenzyme Q10 and α-lipoic acid can attenuate tissue damage (19,20,21).

Apigenin is a flavonoid found in many plants, known for its antioxidant and anti-inflammatory properties. Apigenin attenuates tissue damage via inhibition of oxidative stress and the NOS pathway (9). Apigenin was evaluated for its potential protective effects against I/R injury and was shown to decrease the number of apoptotic cardiomyocytes in I/R injury of the isolated rat heart (22). Another work that investigated the therapeutic effects of apigenin on ischemic stroke revealed that apigenin elevated antioxidant activity and reduced oxidative production (23). In addition, apigenin inhibited apoptosis in a hepatic I/R model (13). Also, apigenin prevented inhibition of the activity of Cu/Zn SOD and increased levels of MDA in I/R-induced myocardium injury (12). The other flavonoid, quercetin, was demonstrated as having an antiapoptotic influence upon experimental ovarian I/R injury (24). In our study, apigenin treatment significantly reduced tissue NOx concentrations and elevated tissue GSH levels in group 4, compared with those in groups 2 and 3. However, MDA and CAT levels were not significantly different among the I/R groups. The results of the present study indicated that, although apigenin partially reduced oxidative stress, it did not ameliorate histopathological damage in the ovarian tissue. Contrary to previous reports, apigenin has no significant protective effect against ovarian I/R injury.

DMSO is a solvent that has anti-inflammatory, antioxidant and antiapoptotic properties (25). Although our results showed that biochemical and histopathological parameters did not differ significantly between groups 2 and 3, DMSO did not affect the results. This result was likely due to the low dose of DMSO we used.

Some reports in the literature demonstrate the effect of therapeutic agents by assessing histopathological damage score on ovarian tissue in experimental ovarian I/R injury. A previous study indicated that selenium therapy reduced the ovarian tissue injury rates in experimental I/R injury (26). In the present study, the histopathological damage scores were elevated in groups 2 and 3 as compared with the sham group. This result might be a consequence of tissue injury caused by not only ischemia but also reperfusion. We observed lower histopathological damage scores in group 4 than in other I/R groups; however, this difference was not statistically significant. Histopathological changes in rat ovary caused by I/R were not restored through apigenin. Tsalkidou et al. (13) have endorsed the inhibitory influence of apigenin on apoptosis in hepatic I/R. Furthermore, a previous study demonstrated that apigenin reduced neuronal cell death in a rat model of diabetes (27). However, it was previously found that apigenin can induce apoptosis in leukaemia HL-60 cells (28). In contrast to the above-mentioned studies, our current investigation showed that apigenin did not improve I/R-induced histopathological changes in ovarian tissue. This result may be associated with insufficient suppression of oxidative stress due to the low dose of apigenin.

AMH level testing is a valuable method of predicting reproductive lifespan and evaluating long-term ovarian function. A previous study, which compared the preoperative and postoperative serum AMH levels recorded that AMH levels were greatly decreased in the torsion and torsion-detorsion groups. Although in the detorsion group, serum AMH concentrations were higher than those in the torsion groups, the discrepancies between the two groups were not statistically significant (5). Another experimental ovarian I/R study indicated that the reductions in preoperative and postoperative serum AMH concentrations were much larger in the detorsion only group than in the detorsion-enoxaparin groups (29). The results of these studies indicate that detorsion alone is insufficient to protect ovarian reserve. The aim of the present study was to examine the influence of apigenin on ovarian reserve. In contrast, we observed that apigenin treatment did not improve serum AMH levels compared with detorsion alone. This result indicates that apigenin treatment combined with detorsion has no additional protective effect on ovarian reserve over detorsion alone.

To the best of our knowledge, this is the first work to investigate the effects of apigenin treatment on I/R-induced ovarian injury. This work has shown that although apigenin was partially effective in reducing oxidative stress, it had no impact on serum AMH levels nor on reduction of ovarian tissue damage as assessed by histological grading. The long-term effect of apigenin treatment, dose adjustment and different modes of administration can be assessed in future studies.

In conclusion, there are no clinically available antioxidant therapies that are effectively used in conjunction with detorsion in the treatment of ovarian torsion to date. Irrespective of previous studies, our study suggests that administration of apigenin has no significant protective effect on ovarian reserve and tissue damage in ovarian I/R injury. We believe that further works should address whether apigenin treatment at different dosages has any protective effect against ovarian I/R injury.

## Figures and Tables

**Table 1:**
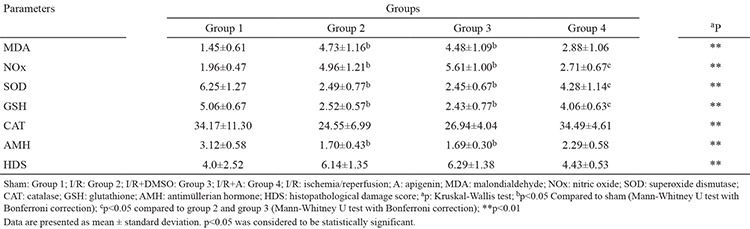
The mean values of biochemical parameters and histopathological damage scores in groups

## References

[1] Houry D, Abbott JT (2001). Ovarian torsion: a fifteen-year review. Ann Emerg Med.

[2] Sasaki KJ, Miller CE (2014). Adnexal torsion: review of the literature. J Minim Invasive Gynecol.

[3] Köleli I (2015). Mean Platelet Volume in Early Diagnosis of Adnexal Torsion. Balkan Med J.

[4] Damigos E, Johns J, Ross J (2012). An update on the diagnosis and management of ovarian torsion. The Obstetrician & Gynaecologist.

[5] Ozler A, Turgut A, Soydinç HE, Sak ME, Evsen MS, Alabalik U, et al (2013). The biochemical and histologic effects of adnexal torsion and early surgical intervention to unwind detorsion on ovarian reserve: an experimental study. Reprod Sci.

[6] Li C, Jackson RM (2002). Reactive species mechanisms of cellular hypoxia-reoxygenation injury. Am J Physiol Cell Physiol.

[7] Baarends WM, Uilenbroek JT, Kramer P, Hoogerbrugge JW, Leeuwen EC, Themmen AP, et al (1995). Anti-müllerian hormone and anti-müllerian hormone type II receptor messenger ribonucleic acid expression in rat ovaries during postnatal development, the estrous cycle, and gonadotropin-induced follicle growth. Endocrinology.

[8] Bonilla-Musoles F, Castillo JC, Caballero O, Pérez-Panades J, Bonilla F, Dolz M, et al (2012). Predicting ovarian reserve and reproductive outcome using antimüllerian hormone (AMH) and antral follicle count (AFC) in patients with previous assisted reproduction technique (ART) failure. Clin Exp Obstet Gynecol.

[9] Huang CS, Lii CK, Lin AH, Yeh YW, Yao HT, Li CC, et al (2013). Protection by chrysin, apigenin, and luteolin against oxidative stress is mediated by the Nrf2-dependent up-regulation of heme oxygenase 1 and glutamate cysteine ligase in rat primary hepatocytes. Arch Toxicol.

[10] Nicholas C, Batra S, Vargo MA, Voss OH, Gavrilin MA, Wewers MD, et al (2007). Apigenin blocks lipopolysaccharide-induced lethality in vivo and proinflammatory cytokines expression by inactivating NF-kappaB through the suppression of p65 phosphorylation. J Immunol.

[11] Liu C, Tu FX, Chen X (2008). [Neuroprotective effects of apigenin on acute transient focal cerebral ischemia-reperfusion injury in rats]. Zhong Yao Cai.

[12] Yang X, Yang J, Hu J, Li X, Zhang X, Li Z (2015). Apigenin attenuates myocardial ischemia/reperfusion injury via the inactivation of p38 mitogen‑activated protein kinase. Mol Med Rep.

[13] Tsalkidou EG, Tsaroucha AK, Chatzaki E, Lambropoulou M, Papachristou F, Trypsianis G, et al (2014). The effects of apigenin on the expression of Fas/FasL apoptotic pathway in warm liver ischemia-reperfusion injury in rats. Biomed Res Int.

[14] Ohkawa H, Ohishi N, Yagi K (1979). Assay for lipid peroxides in animal tissues by thiobarbituric acid reaction. Anal Biochem.

[15] Sun Y, Oberley LW, Li Y (1988). A simple method for clinical assay of superoxide dismutase. Clin Chem.

[16] Beutler E, Duron O, Kelly MB (1963). Improved method for the determination of blood glutathione. J Lab Clin Med.

[17] Guven S, Muci E, Unsal MA, Yulug E, Alver A, Kadioglu Duman M, et al (2010). The effects of carbon dioxide pneumoperitoneum on ovarian blood flow, oxidative stress markers, and morphology during laparoscopy: a rabbit model. Fertil Steril.

[18] Bulger EM, Maier RV (2001). Antioxidants in critical illness. Arch Surg.

[19] Cosar E, Sahin FK, Köken G, Toy H, Basarali K, Büyükbas S (2007). The protective effect of alpha-lipoic acid in experimental ovarian ischaemia-reperfusion injury. Aust N Z J Obstet Gynaecol.

[20] Bakan V, Ciralik H, Tolun FI, Atli Y, Mil A, Oztürk S (2009). Protective effect of erythropoietin on torsion/detorsion injury in rat model. J Pediatr Surg.

[21] Ozler A, Turgut A, Görük NY, Alabalik U, Basarali MK, Akdemir F (2013). Evaluation of the protective effects of CoQ₁₀ on ovarian I/R injury: an experimental study. Gynecol Obstet Invest.

[22] Hu J, Li Z, Xu LT, Sun AJ, Fu XY, Zhang L, et al (2015). Protective effect of apigenin on ischemia/reperfusion injury of the isolated rat heart. Cardiovasc Toxicol.

[23] Cai M, Ma Y, Zhang W, Wang S, Wang Y, Tian L, et al (2016). Apigenin-7-O-β-D-(-6''-p-coumaroyl)-Glucopyranoside Treatment Elicits Neuroprotective Effect against Experimental Ischemic Stroke. Int J Biol Sci.

[24] Gencer M, Karaca T, Güngör AN, Hacıvelioğlu SÖ, Demirtaş S, Turkon H, et al (2014). The protective effect of quercetin on IMA levels and apoptosis in experimental ovarian ischemia-reperfusion injury. Eur J Obstet Gynecol Reprod Biol.

[25] Santos NC, Figueira-Coelho J, Martins-Silva J, Saldanha C (2003). Multidisciplinary utilization of dimethyl sulfoxide: pharmacological, cellular, and molecular aspects. Biochem Pharmacol.

[26] Bozkurt S, Arikan DC, Kurutas EB, Sayar H, Okumus M, Coskun A, et al (2012). Selenium has a protective effect on ischemia/reperfusion injury in a rat ovary model: biochemical and histopathologic evaluation. J Pediatr Surg.

[27] Mao XY, Yu J, Liu ZQ, Zhou HH (2015). Apigenin attenuates diabetes-associated cognitive decline in rats via suppressing oxidative stress and nitric oxide synthase pathway. Int J Clin Exp Med.

[28] Wang IK, Lin-Shiau SY, Lin JK (1999). Induction of apoptosis by apigenin and related flavonoids through cytochrome c release and activation of caspase-9 and caspase-3 in leukaemia HL-60 cells. Eur J Cancer.

[29] Kaya C, Turgut H, Cengiz H, Turan A, Ekin M, Yaşar L (2014). Effect of detorsion alone and in combination with enoxaparin therapy on ovarian reserve and serum antimüllerian hormone levels in a rat ovarian torsion model. Fertil Steril.

